# Long-term TSH suppression in metabolically unhealthy survivors of differentiated thyroid cancer: a cardiovascular perspective

**DOI:** 10.3389/fcvm.2026.1924477

**Published:** 2026-08-19

**Authors:** Jiangtao Chai, Xiaoting Cai, Yanan Zhang, Qidong Tan, Wei Qu, Ying Qian

**Affiliations:** Department of Endocrinology, 960 Hospital of People’s Liberation Army, Jinan, China

**Keywords:** atrial fibrillation, cardiovascular risk, differentiated thyroid cancer, metabolic syndrome, TSH suppression

## Abstract

Differentiated thyroid cancer (DTC) is associated with favorable long-term survival in most patients, shifting clinical priorities from recurrence prevention alone toward survivorship care and competing cardiovascular risk. Thyroid-stimulating hormone (TSH) suppression with levothyroxine remains biologically plausible and clinically important in selected patients with high-risk disease, biochemical incomplete response, or persistent structural disease. However, prolonged exogenous subclinical hyperthyroidism may increase heart rate, atrial arrhythmogenicity, myocardial workload, vascular stress, and cardiovascular vulnerability. This issue is particularly relevant in metabolically unhealthy survivors, including those with obesity, insulin resistance, diabetes, hypertension, dyslipidemia, metabolic syndrome, chronic kidney disease, or established atherosclerotic cardiovascular disease. In these patients, TSH suppression may interact with a pre-existing pro-arrhythmic, pro-inflammatory, and pro-atherogenic substrate, although direct evidence that metabolic phenotype modifies suppression-related cardiovascular risk is currently lacking. This narrative review examines the evolving benefit-harm balance of long-term TSH suppression in DTC survivorship from a cardiovascular perspective. We discuss the oncologic rationale and uncertainty of persistent suppression, cardiovascular liabilities of chronic low TSH exposure, the potential role of cardiometabolic dysfunction as a modifier of treatment tolerance, treatment-related modifiers including radioiodine preparation and multikinase inhibitors, and a hypothesis-generating metabolism-informed framework for TSH target selection. The proposed framework integrates recurrence risk, dynamic response to therapy, cardiometabolic phenotype, cumulative suppression exposure, and cardiovascular reserve, but should be regarded as a hypothesis-generating model that requires prospective validation before it can guide TSH targets.

## 1 Introduction

Differentiated thyroid cancer (DTC), predominantly comprising papillary and follicular thyroid carcinoma, is associated with favorable long-term survival in most patients after appropriate initial treatment (1, 2). Consequently, DTC care increasingly extends beyond recurrence prevention to long-term survivorship, in which endocrine treatment, aging, cardiometabolic comorbidity, and competing non-cancer mortality may interact over several decades.

Levothyroxine after thyroidectomy serves both physiological hormone replacement and pharmacological suppression of thyroid-stimulating hormone (TSH). The oncologic rationale for suppression is based on the trophic effect of TSH on thyroid follicular cells and the possibility that persistent stimulation may promote residual tumor growth (3). More intensive suppression may remain appropriate in patients with persistent structural disease, biochemical incomplete response, or other high-risk features. However, prolonged treatment can also produce an exogenous subclinical hyperthyroidism-like state, with potential cardiovascular, skeletal, and metabolic consequences.

The principal clinical uncertainty is therefore not whether TSH suppression has a role, but which patients continue to derive sufficient oncologic benefit to justify its intensity and duration. A recent systematic review and meta-analysis found no clear survival advantage from suppression in intermediate- and high-risk DTC and reported an increased burden of secondary complications, although the underlying evidence was heterogeneous and predominantly observational (4). An accompanying editorial similarly emphasized persistent uncertainty regarding optimal intensity, duration, and patient selection (5). These findings support dynamic reassessment rather than routine lifelong continuation of the initial postoperative target.

Cardiovascular risk is an important component of this reassessment. Meta-analyses have reported associations between DTC treatment or long-term TSH suppression and atrial fibrillation, higher heart rate, blood pressure changes, and other cardiovascular outcomes (6, 7). A broader synthesis also identified possible excess risks of atrial fibrillation and cardiovascular mortality among DTC survivors (8). These findings should not be interpreted as proof that TSH suppression itself causes cardiovascular events, because cancer severity, levothyroxine exposure, radioiodine treatment, age, pre-existing disease, surveillance intensity, and confounding by indication may contribute to the observed associations.

Cardiometabolic dysfunction may further influence treatment tolerance. Insulin resistance, dysglycemia, obesity, and hypertension have been associated with thyroid cancer risk, while metabolic syndrome and metabolically unhealthy obesity have also been linked to thyroid cancer incidence or prognosis (9–11). These studies do not demonstrate that metabolic phenotype modifies the cardiovascular effects of TSH suppression. They nevertheless identify a survivor population with an established background of vascular, myocardial, and arrhythmic vulnerability. This review therefore examines whether cardiometabolic dysfunction could alter the benefit-harm balance of prolonged suppression when interpreted alongside recurrence risk and dynamic response to therapy. The proposed metabolism-informed framework is hypothesis-generating and requires prospective validation before it can be used as a treatment algorithm.

For this narrative review, we searched PubMed from inception to June 26, 2026, using combinations of the following terms: “differentiated thyroid cancer,” “papillary thyroid cancer,” “thyroid-stimulating hormone suppression,” “TSH suppression,” “levothyroxine,” “subclinical hyperthyroidism,” “cardiovascular outcomes,” “atrial fibrillation,” “cardiovascular mortality,” “metabolic syndrome,” “insulin resistance,” “obesity,” “metabolically unhealthy obesity,” “diabetes,” “hypertension,” “dyslipidemia,” “radioactive iodine,” “radioiodine,” “thyroid hormone withdrawal,” “lenvatinib,” “tyrosine kinase inhibitor,” “triiodothyronine,” “T3,” “FT3/FT4 ratio,” “deiodinase,” “DIO1,” “brain natriuretic peptide,” “BNP,” and “NT-proBNP.” Only peer-reviewed articles published in English were included. Priority was given to clinical guidelines, systematic reviews, meta-analyses, large cohort studies, and mechanistic or translational studies relevant to DTC survivorship, TSH suppression, cardiometabolic risk, peripheral thyroid hormone metabolism, and cardiovascular outcomes. Additional references were identified by screening the reference lists of key articles. Because this was a narrative review rather than a systematic review, study selection was based on relevance to the scope of the review, and formal risk-of-bias assessment and quantitative evidence synthesis were not performed.

## 2 Reframing TSH suppression in the era of long-term DTC survivorship

### 2.1 From recurrence prevention to survivorship medicine

TSH suppression has historically occupied a central position in postoperative DTC management because it offers a biologically plausible and clinically actionable strategy to reduce trophic stimulation of residual thyroid cancer cells. In this conventional framework, levothyroxine is not only a replacement therapy after thyroidectomy, but also an oncologic intervention intended to maintain serum TSH below a predefined threshold. This approach remains clinically reasonable in patients with persistent structural disease, biochemical incomplete response, or high-risk clinicopathological features, in whom the risk of progression may justify more intensive endocrine suppression (2, 3). The therapeutic objective is therefore neither universal biochemical normalization nor maximal TSH suppression in all survivors. It is to use the least intensive degree of suppression that remains appropriate for the patient's current oncologic status while avoiding unnecessary thyroid hormone excess (1–3).

However, the meaning of TSH suppression changes when DTC is viewed as a long-term survivorship condition. Many patients, particularly those with low-risk or intermediate-risk disease who achieve an excellent response after initial treatment, face a relatively low probability of disease-specific mortality but a prolonged exposure to levothyroxine, surveillance, and age-related cardiometabolic comorbidities. In this setting, the key clinical question is no longer simply whether TSH suppression can reduce recurrence, but whether a given degree of suppression remains justified after the expected oncologic benefit has diminished. This distinction is important because the cumulative harm of suppression may increase over time, whereas the absolute recurrence-prevention benefit may decrease after durable remission. In low-risk or excellent-response survivors, this balance may favor TSH values within or closer to the physiological range, whereas persistent high-risk disease may still justify more intensive suppression.

Contemporary guidelines have increasingly recognized this shift by recommending individualized TSH targets according to recurrence risk, treatment response, and adverse-effect risk rather than universal lifelong suppression (1, 2). This evolution reflects a broader transition in DTC care: the goal is not maximal treatment intensity, but calibrated treatment intensity. For survivors with cardiometabolic dysfunction, this distinction may be particularly relevant because baseline cardiovascular reserve may already be reduced. However, direct evidence that metabolic phenotype modifies the cardiovascular effects of long-term TSH suppression remains unavailable. Therefore, TSH suppression should be conceptualized as a modifiable long-term exposure whose intensity and duration require periodic reassessment, rather than as a static extension of initial cancer therapy.

### 2.2 Dynamic risk stratification and the changing need for suppression

Initial postoperative risk stratification remains essential, but it is incomplete because recurrence risk is not fixed at diagnosis. Dynamic risk stratification addresses this limitation by incorporating response-to-therapy variables during follow-up, including serum thyroglobulin, anti-thyroglobulin antibodies, structural imaging, and clinical disease status. In a pivotal study of 588 adult patients with follicular cell-derived thyroid cancer treated with total thyroidectomy and radioactive iodine remnant ablation, Tuttle and colleagues demonstrated that the initial ATA recurrence risk estimates could be substantially modified by response to therapy during the first two years of follow-up (12). An excellent response markedly reduced the likelihood of persistent structural disease or recurrence across initial risk categories, whereas incomplete response increased subsequent risk, supporting a dynamic rather than static approach to follow-up and treatment intensity.

This concept has practical implications for TSH suppression. A patient who initially required moderate or strong suppression because of intermediate-risk features may no longer have the same benefit-harm profile after achieving an excellent response. Conversely, a patient initially considered lower risk but later showing biochemical or structural incomplete response may require more intensive therapy. Thus, response-to-therapy classification provides a clinical mechanism for adjusting TSH targets over time. It also creates the space to incorporate non-oncologic risks, including atrial fibrillation risk, osteoporosis risk, diabetes, hypertension, dyslipidemia, obesity, and established cardiovascular disease.

Importantly, dynamic risk stratification is not limited to patients who receive radioactive iodine. In patients treated with lobectomy or total thyroidectomy without radioactive iodine, modified response-to-therapy definitions have also been validated. Momesso and colleagues evaluated 507 adults with DTC treated without radioactive iodine and found that recurrent or persistent structural disease was strongly associated with response category; no structural evidence of disease was observed in patients with excellent response, whereas structural incomplete response was associated with persistent or recurrent disease (13). Park and colleagues similarly showed that a dynamic risk stratification system could predict recurrent or persistent disease in patients treated without radioactive iodine remnant ablation, including those who underwent lobectomy or total thyroidectomy (14). These findings are relevant because modern DTC care increasingly uses more selective surgery and radioactive iodine strategies, making flexible long-term risk assessment essential.

For the present review, the most important implication is that TSH suppression should follow the patient's evolving risk state. If cancer risk is dynamic, endocrine treatment intensity should also be dynamic. A fixed long-term TSH target may be clinically inappropriate when a survivor's tumor status has improved but metabolic and cardiovascular risk has worsened. This situation is common in real-world survivorship: patients age, gain weight, develop hypertension or diabetes, and accumulate atherosclerotic risk while remaining free of thyroid cancer recurrence. Dynamic risk stratification therefore provides the oncologic basis on which evolving cardiometabolic vulnerability may be considered. Whether metabolic phenotype should directly alter TSH targets, however, remains untested and requires prospective validation.

### 2.3 The unresolved benefit-harm threshold

The evidence base supporting TSH suppression is heterogeneous and largely observational. Earlier registry data suggested that greater TSH suppression was more commonly used in higher-risk patients and that the need for strong suppression was less convincing in low-risk disease. In the National Thyroid Cancer Treatment Cooperative Registry, Cooper and colleagues found that disease stage, age, and radioiodine therapy were associated with progression, while the degree of TSH suppression did not clearly support the need for greater suppression in low-risk patients; the possible value of suppression appeared more relevant in high-risk disease, but uncertainty remained (15). This older evidence remains important because it already anticipated the current controversy: suppression may not have a uniform benefit across risk groups.

More recent data have further complicated the assumption that stronger suppression necessarily improves clinically meaningful outcomes. In a multicenter cohort study of 867 patients with intermediate- and high-risk DTC treated with total thyroidectomy and radioactive iodine, Klubo-Gwiezdzinska and colleagues found that thyrotropin suppression was not associated with improved progression-free survival or overall survival during landmark analyses (16). A subsequent systematic review and meta-analysis also suggested that TSH suppression in intermediate- and high-risk DTC may not clearly improve survival outcomes and may increase secondary complications, although the evidence was limited and heterogeneous (4). These findings do not prove that TSH suppression should be abandoned. Rather, they indicate that the benefit threshold is uncertain and that patient selection is central.

The low-risk and excellent-response populations are particularly important for avoiding overtreatment. In ATA low- and intermediate-risk patients, Wang and colleagues reported that TSH suppression increased the risk of postoperative osteoporosis without decreasing recurrence, reinforcing the principle that treatment harm can become clinically dominant when baseline recurrence risk is low (17). Although this study focused on skeletal rather than cardiovascular harm, its conceptual relevance is direct: long-term suppression can produce clinically meaningful adverse effects without a proportional oncologic gain in indolent disease. A similar logic should be applied to cardiometabolic harm, especially in survivors with obesity, diabetes, hypertension, dyslipidemia, atrial fibrillation susceptibility, or established atherosclerotic disease.

Taken together, the available DTC literature supports a restrained and response-adapted interpretation of long-term TSH suppression. Stronger suppression remains most defensible when the expected oncologic benefit is high, particularly in structural incomplete response or persistent high-risk disease. In low-risk or excellent-response survivors, its incremental benefit becomes less certain as durable remission is established. Cardiometabolic dysfunction may further reduce treatment tolerance, but direct evidence of an interaction between metabolic phenotype and suppression-related cardiovascular outcomes is currently unavailable. The metabolism-informed approach proposed here should therefore be viewed as a hypothesis for clinical testing rather than an established basis for selecting TSH targets.

## 3 Cardiovascular liabilities of chronic exogenous subclinical hyperthyroidism

### 3.1 Electrophysiological consequences: atrial fibrillation as the sentinel outcome

Long-term TSH suppression should be interpreted as a chronic cardiovascular exposure rather than only as an endocrine oncology strategy. In most DTC survivors receiving suppressive levothyroxine therapy, serum TSH is intentionally reduced below the physiological range, FT4 is commonly maintained within or toward the upper reference range, and circulating T3 varies partly according to peripheral conversion. This biochemical pattern resembles exogenous subclinical hyperthyroidism and can influence cardiac electrophysiology, autonomic tone, myocardial workload, and vascular function. Therefore, even when overt thyrotoxicosis is absent, persistent TSH suppression may create a sustained cardiovascular stress state, particularly in patients with limited cardiometabolic reserve (3, 6–8).

Atrial fibrillation is the most clinically recognizable cardiovascular outcome in this setting. A meta-analysis of cardiovascular outcomes in thyroid cancer patients treated with thyroidectomy found that DTC was associated with increased risks of atrial fibrillation, coronary artery disease, cerebrovascular accidents, and all-cause mortality; it also reported higher heart rate, higher diastolic blood pressure, greater left ventricular mass index, and altered diastolic filling parameters in DTC patients compared with controls (18). These findings are not sufficient to attribute all cardiovascular excess risk to TSH suppression, because thyroidectomy, radioiodine exposure, baseline comorbidities, surveillance intensity, and cancer severity may contribute. Nevertheless, they identify a cardiovascular safety signal in treated DTC survivors, without isolating the independent contribution of TSH suppression.

The electrophysiological plausibility is strong. Thyroid hormone excess can increase sinus rate, enhance beta-adrenergic responsiveness, shorten atrial refractory periods, and promote atrial ectopy. In metabolically healthy younger survivors, these changes may remain subclinical or clinically tolerable. In metabolically unhealthy survivors, however, the same endocrine exposure may occur on the background of hypertension, obesity, insulin resistance, sleep apnea, left atrial enlargement, systemic inflammation, and vascular stiffness. This background substrate can lower the threshold for atrial fibrillation, making rhythm-related risk a central issue when long-term TSH targets are selected.

### 3.2 Hemodynamic and vascular consequences

The cardiovascular effect of TSH suppression extends beyond atrial electrophysiology. Chronic exogenous subclinical hyperthyroidism may increase resting heart rate, cardiac output, myocardial oxygen demand, and pulse pressure, while also affecting ventricular relaxation and arterial compliance. These changes are usually modest when considered individually, but their cumulative impact may become clinically meaningful over years of exposure, especially in older patients or in those with diabetes, hypertension, coronary artery disease, or chronic kidney disease.

DTC-specific data support this concern. A meta-analysis focused on incident atrial fibrillation in patients with DTC reported a higher incidence of atrial fibrillation compared with controls, reinforcing AF as a sentinel cardiovascular endpoint in this population (19). In a cohort study, Klein Hesselink and colleagues also reported an increased risk of atrial fibrillation after treatment for differentiated thyroid carcinoma (20). These observations align with broader thyroid literature: in pooled individual participant data, endogenous subclinical hyperthyroidism was associated with increased risks of total mortality, coronary heart disease mortality, and incident atrial fibrillation, with the highest risks generally observed at lower TSH concentrations (21). A large individual participant data analysis further suggested that higher free thyroxine levels, even within the reference range, were associated with increased risk of incident atrial fibrillation (22).

These general-population data are relevant to DTC survivorship because suppressive levothyroxine therapy intentionally shifts patients toward a low-TSH state for prolonged periods. However, DTC survivors differ from typical endogenous subclinical hyperthyroidism cohorts in important ways. They are exposed to clinician-directed levothyroxine dosing, repeated monitoring, changing TSH targets, and varying degrees of cancer-related treatment burden. Therefore, extrapolation from endogenous subclinical hyperthyroidism should be cautious. The more appropriate interpretation is that general thyroid-cardiovascular evidence provides biological plausibility, while DTC-specific cohorts and meta-analyses provide disease-contextual evidence.

### 3.3 Cumulative exposure and competing risks

The cardiovascular consequences of TSH suppression are likely determined by cumulative exposure rather than by a single TSH measurement. Clinically relevant variables include the depth of TSH suppression, duration of suppression, levothyroxine dose, age at exposure, sex, baseline cardiovascular disease, blood pressure control, glycemic status, lipid burden, renal function, and competing cancer vs. non-cancer mortality risks. This cumulative-exposure perspective is especially important for DTC because many survivors remain on levothyroxine for decades.

Several mechanistic and imaging studies support the possibility that long-term suppressive therapy affects cardiovascular structure and function. Shargorodsky and colleagues reported that long-term thyrotropin-suppressive therapy with levothyroxine impaired small and large artery elasticity and increased left ventricular mass in patients with thyroid carcinoma (23). Abdulrahman and colleagues showed that both exogenous subclinical hyperthyroidism and short-term overt hypothyroidism affected myocardial strain in patients with DTC, suggesting that thyroid hormone perturbation can influence subclinical myocardial mechanics (24). Although these studies were relatively small and should not be overinterpreted as definitive event-level evidence, they provide mechanistic support for the hemodynamic and myocardial pathways through which long-term thyroid hormone manipulation may affect cardiovascular vulnerability.

Event-level observational data are also clinically relevant. Klein Hesselink and colleagues reported increased long-term cardiovascular mortality in patients with differentiated thyroid carcinoma and found that lower TSH levels were associated with higher cardiovascular mortality risk (25). In a Korean nationwide cohort study, Suh and colleagues found that thyroid cancer patients taking levothyroxine had increased risks of coronary heart disease and ischemic stroke, with higher levothyroxine dosage associated with greater risk (26). Pajamäki and colleagues similarly evaluated long-term cardiovascular morbidity and mortality in DTC patients and highlighted the need for cardiovascular attention during follow-up (27). A Swedish nationwide study of 6900 DTC patients also reported increased hospitalization for atrial fibrillation and, among women, increased hospitalization for cerebrovascular disease compared with the general population (28).

These findings require cautious interpretation. Patients selected for deeper or more prolonged TSH suppression frequently have more advanced, persistent, or recurrent disease and may also receive greater cumulative radioiodine exposure, systemic therapy, and surveillance. Cancer severity, age, levothyroxine dose, baseline obesity, pre-existing cardiovascular disease, renal dysfunction, and healthcare utilization may therefore contribute to the observed associations. Confounding by indication is particularly important because the clinical reason for stronger suppression may itself be associated with worse long-term outcomes. Levothyroxine dose is also an imperfect surrogate for biological exposure, while many datasets lack serial TSH, FT4, treatment-response status, adherence, and time-updated cardiovascular risk factors. Accordingly, current observational studies support a cardiovascular safety signal and a rationale for individualized reassessment, but they do not establish that TSH suppression independently causes cardiovascular events (4, 6–8, 18–28). The major evidence domains supporting this benefit-harm interpretation are summarized in Table 1.

**Table 1:** Evidence domains informing a proposed metabolism-informed cardiovascular perspective on long-term TSH suppression in DTC survivorship.

Evidence	Core evidence	Implication	Key limitations	References
TSH suppression is useful but should be individualized	ATA guidelines and classic reviews support TSH suppression in selected DTC patients, especially those with higher recurrence risk or persistent disease, but also recognize adverse-effect concerns.	TSH suppression should be treated as a benefit–harm intervention rather than a uniform lifelong strategy.	Recommendations rely partly on observational evidence and expert consensus.	(1–3)
Recurrence risk changes during follow-up	Dynamic risk stratification studies show that response to therapy substantially modifies initial recurrence-risk estimates, including in patients treated without radioiodine.	Long-term TSH targets should be reassessed after excellent, indeterminate, biochemical incomplete, or structural incomplete response.	Most studies focus on oncologic outcomes, not cardiovascular outcomes.	(12–14)
Strong suppression has uncertain benefit in selected survivors	Registry, cohort, systematic review, and meta-analysis evidence suggest that strong long-term suppression may not clearly improve survival or recurrence outcomes in all low-risk, intermediate-risk, or excellent-response patients.	The need for chronic strong suppression is weakest when recurrence risk is low and treatment response is excellent.	Heterogeneous study design, variable TSH definitions, and limited randomized evidence.	(4, 5, 15–17)
DTC survivorship and TSH suppression are linked to cardiovascular risk signals	Meta-analyses and cohort studies report increased atrial fibrillation, cardiovascular morbidity, cardiovascular mortality, coronary heart disease, ischemic stroke, or cardiovascular hospitalization in DTC survivors or patients receiving suppressive therapy.	Cardiovascular risk should be considered when deciding the intensity and duration of TSH suppression.	Residual confounding by age, disease severity, levothyroxine dose, radioiodine exposure, and baseline comorbidity.	(6–8, 18–28)
Metabolic unhealthiness identifies a vulnerable cardiovascular substrate	Metabolic syndrome, insulin resistance, diabetes, obesity, dyslipidemia, and metabolically unhealthy obesity are associated with thyroid cancer risk and, more importantly, with increased cardiovascular risk.	Cardiometabolic dysfunction may mark reduced baseline cardiovascular reserve; direct modification of suppression-related risk has not been demonstrated.	Thyroid cancer associations may be affected by detection bias and residual confounding.	(9–11, 29–37)
Mechanistic convergence is biologically plausible	Thyroid hormone excess may increase heart rate, adrenergic tone, myocardial workload, and arrhythmogenicity, while metabolic dysfunction promotes atrial remodeling, endothelial dysfunction, vascular inflammation, and atherosclerotic stress.	Biological plausibility suggests potentially lower treatment tolerance, but the proposed interaction requires DTC-specific longitudinal validation.	Mechanistic links require more DTC-specific longitudinal validation.	(38–44)
Other DTC treatments may add cardiovascular burden	Radioiodine itself has not consistently shown independent long-term cardiovascular harm, but thyroid hormone withdrawal can worsen metabolic parameters, and VEGFR-targeted therapy can cause hypertension and vascular toxicity.	Cardiovascular risk in DTC survivorship is cumulative and treatment-layered.	Applies variably across patients; TKI-related risk mainly concerns advanced or radioiodine-refractory DTC.	(47–54)
Cardiovascular survivorship care should be integrated into DTC follow-up	Cardio-oncology, cardiovascular prevention, and atrial fibrillation guidelines support baseline risk assessment, risk-factor modification, and rhythm surveillance in vulnerable cancer survivors.	Cardiovascular prevention should complement guideline-based oncologic assessment; the use of metabolic phenotype to select TSH targets remains unvalidated.	Existing risk tools are not designed specifically for long-term suppressive levothyroxine exposure in DTC.	(55–62)

## 4 Cardiometabolic dysfunction as a potential modifier of DTC survivorship risk

### 4.1 Beyond BMI: defining the metabolically unhealthy survivor

Metabolic unhealthiness in DTC survivors should be defined by overall cardiometabolic risk burden rather than by body mass index alone. The harmonized definition of metabolic syndrome identifies a cluster of central obesity, elevated blood pressure, raised triglycerides, reduced high-density lipoprotein cholesterol, and impaired fasting glucose, with the presence of at least three components generally used to define metabolic syndrome (29). This framework is clinically useful because it distinguishes excess body weight from the broader metabolic milieu that determines vascular, myocardial, and arrhythmic vulnerability. A patient with normal BMI but diabetes, hypertension, and atherogenic dyslipidemia may have greater cardiovascular vulnerability than a patient with obesity but preserved metabolic health. For the purposes of this review, cardiometabolic dysfunction is treated as a risk-burden construct rather than a binary diagnosis that requires fulfillment of formal metabolic syndrome criteria. Established diabetes, hypertension, atherogenic dyslipidemia, chronic kidney disease, prior atrial fibrillation, heart failure, or atherosclerotic cardiovascular disease may each indicate clinically relevant vulnerability. Central obesity should be interpreted together with blood pressure, glucose metabolism, lipid profile, sleep-disordered breathing, and evidence of target-organ disease. Obesity without metabolic abnormalities should not automatically be considered sufficient to alter TSH management, and no single metabolic diagnosis independently mandates relaxation of oncologically indicated suppression.

The cardiovascular relevance of this phenotype is well established. In a systematic review and meta-analysis, metabolic syndrome was associated with an approximately two-fold increase in cardiovascular outcomes and a significant increase in all-cause mortality (30). This background risk is directly relevant to DTC survivorship, because long-term TSH suppression may add hemodynamic and electrophysiological stress to an already unfavorable cardiometabolic substrate. Therefore, metabolically unhealthy DTC survivors should be understood as patients with central obesity, insulin resistance, prediabetes or diabetes, hypertension, dyslipidemia, metabolic syndrome, metabolic dysfunction-associated steatotic liver disease, chronic kidney disease, atrial fibrillation susceptibility, or established atherosclerotic cardiovascular disease.

This broader definition is intentional: TSH suppression is applied to individual survivors rather than to a single metabolic diagnosis, and cardiovascular vulnerability may evolve over years of follow-up. In this context, metabolic phenotype should be regarded as a marker of baseline cardiovascular vulnerability that may influence treatment tolerance. It has not been established as an effect modifier of suppression-related cardiovascular outcomes (29, 30).

### 4.2 Metabolic dysfunction and thyroid cancer: association, bias, and biology

The association between metabolic dysfunction and thyroid cancer has been reported in systematic review evidence, although causal interpretation remains complex. Insulin resistance, dysglycemia, increased body mass index, and hypertension have been associated with thyroid cancer risk, while metabolic syndrome and its components may also be related to thyroid cancer risk, aggressiveness, and prognosis (9, 10). Several specific metabolic exposures support this association. In the Sister Study, obesity and obesity-related metabolic conditions were prospectively evaluated in relation to thyroid cancer risk among women, while meta-analysis evidence has also linked diabetes mellitus and insulin resistance with increased thyroid cancer risk (31–33).

The temporal and qualitative features of metabolic dysfunction may be more informative than a single BMI measurement. In a population-based cohort study of young adults, cumulative exposure to metabolic syndrome across repeated health examinations was associated with progressively increased thyroid cancer risk, suggesting that metabolic dysfunction should be viewed as a cumulative exposure rather than a static baseline characteristic (34). Metabolic obesity phenotypes further support this view: cohort evidence and systematic exploration indicate that metabolically unhealthy obesity is associated with thyroid cancer risk, whereas adiposity alone may be less informative than the metabolic quality of adiposity (11, 35). Obesity itself remains relevant, with recent cohort meta-analysis evidence supporting an association with thyroid cancer risk, but it should be interpreted alongside glycemic status, blood pressure, lipid profile, inflammatory burden, and cardiovascular disease rather than in isolation (36).

Detection bias remains an important limitation. Individuals with obesity, diabetes, hypertension, or metabolic syndrome may undergo more frequent clinical evaluation, laboratory testing, and imaging, increasing the likelihood of detecting small or indolent papillary thyroid cancers. Nevertheless, detection bias does not fully exclude biological relevance, because insulin resistance, chronic inflammation, adipokine imbalance, oxidative stress, altered thyroid hormone dynamics, and insulin-like growth factor signaling provide plausible mechanistic links between metabolic dysfunction and thyroid carcinogenesis (9, 10, 33). For the present review, the key point is not whether metabolic dysfunction is a definitive cause of DTC, but that it identifies a survivor population with heightened cardiovascular vulnerability during long-term endocrine therapy.

### 4.3 Metabolic phenotype as a potential cardiovascular risk amplifier after cancer treatment

After DTC treatment, metabolic dysfunction acquires a different clinical meaning. Before diagnosis, obesity, diabetes, insulin resistance, and metabolic syndrome may be discussed as potential contributors to thyroid cancer incidence, progression, or detection. During survivorship, however, these same factors become determinants of competing cardiovascular risk, long-term treatment tolerance, and vulnerability to endocrine overtreatment (30). This transition is central to a cardiovascular interpretation of TSH suppression: chronic suppression may increase heart rate, adrenergic sensitivity, myocardial workload, and atrial arrhythmogenicity, while metabolic dysfunction may lower the threshold at which these endocrine effects become clinically harmful (18–22).

Metabolic health is also dynamic. In the Nurses’ Health Study, transition from a metabolically healthy to a metabolically unhealthy phenotype was associated with increased cardiovascular disease risk across BMI categories during long-term follow-up (37). This observation is highly relevant to DTC survivorship because patients may remain free of thyroid cancer recurrence while gradually developing diabetes, hypertension, dyslipidemia, weight gain, atrial vulnerability, or subclinical cardiovascular disease. Therefore, a TSH target selected shortly after thyroidectomy may not retain the same benefit-harm profile years later, particularly in low-risk or excellent-response survivors in whom the absolute oncologic benefit of strong long-term suppression may be limited (4, 17).

Metabolic phenotype should therefore be considered a potential marker of treatment tolerance rather than a proven modifier of suppression-related risk. A younger survivor without hypertension, diabetes, renal disease, structural heart disease, or arrhythmic susceptibility may tolerate mild suppression differently from an older survivor with several of these conditions. However, current evidence does not establish that the cardiovascular effect of a given TSH level differs causally between these phenotypes. The practical implication is not that metabolic disease should override oncologic status, but that recurrence risk, treatment response, cardiometabolic trajectory, and cumulative suppression exposure should be reassessed together (1, 2).

## 5 Pathophysiological convergence: why TSH suppression may be less tolerable in metabolically unhealthy survivors

### 5.1 Atrial substrate and trigger convergence

The atrial risk of long-term TSH suppression is best understood through a substrate-trigger model. Thyroid hormone has direct cardiovascular actions, including effects on heart rate, myocardial contractility, systemic vascular resistance, and cardiac electrophysiology (38). Even subclinical thyroid dysfunction can be associated with increased heart rate, atrial arrhythmias, increased left ventricular mass, impaired ventricular relaxation, and reduced exercise performance (39). In DTC survivors receiving suppressive levothyroxine therapy, persistent low TSH may therefore function as an arrhythmogenic trigger even when overt thyrotoxicosis is absent.

Metabolic dysfunction can provide the vulnerable atrial substrate on which this trigger acts. Obesity is linked to atrial fibrillation through left atrial enlargement, increased cardiac loading, autonomic imbalance, systemic inflammation, oxidative stress, and neurohormonal activation (40). Diabetes and obesity are also closely connected to AF through inflammatory and oxidative pathways that promote atrial electrical and structural remodeling (41). Epicardial adipose tissue may further contribute to AF by local inflammatory, adipokine-mediated, fibrotic, and autonomic interactions with the atrial myocardium (42).

This convergence is particularly relevant to metabolically unhealthy DTC survivors. In these patients, chronic TSH suppression may not need to create a new arrhythmic substrate *de novo*; rather, it may lower the threshold for AF in an atrium already remodeled by hypertension, central obesity, insulin resistance, sleep apnea, diabetes, or epicardial adiposity. This interpretation is consistent with DTC-specific evidence linking thyroid cancer treatment and long-term suppression with increased AF risk, and with general thyroid literature showing higher AF risk at lower TSH or higher free thyroxine levels (18–22).

### 5.2 Vascular inflammation and atherosclerotic stress

Metabolically unhealthy survivors also have a vascular phenotype that may be less tolerant of chronic endocrine stress. Metabolic syndrome is associated with increased cardiovascular outcomes and mortality, reflecting the combined effects of hypertension, dyslipidemia, impaired glucose metabolism, central adiposity, and chronic inflammation (30). Atherosclerosis is now understood as an inflammatory disease of the arterial wall rather than a passive lipid-storage disorder (43). Diabetes and insulin resistance further impair endothelial function, reducing the anti-atherogenic capacity of the vascular endothelium and promoting vascular dysfunction (44).

TSH suppression may not directly cause atherosclerosis in all patients, but it can add hemodynamic stress to an already vulnerable circulation. Thyroid hormone excess increases heart rate, myocardial contractility, and cardiac output, and can reduce systemic vascular resistance while increasing myocardial oxygen demand (38). Subclinical hyperthyroidism has also been linked to increased heart rate, atrial arrhythmias, ventricular remodeling, impaired relaxation, and cardiovascular death risk (39). In a metabolically unhealthy survivor with endothelial dysfunction, hypertension, dyslipidemia, or diabetes, these effects may increase ischemic vulnerability even without direct plaque acceleration.

This distinction is important for the cardiovascular framing of DTC survivorship. The question is not whether suppressive levothyroxine is an independent atherogenic therapy equivalent to diabetes or dyslipidemia. The more clinically relevant question is whether long-term low TSH amplifies the cardiovascular consequences of pre-existing metabolic disease. Observational DTC studies reporting higher cardiovascular mortality, coronary heart disease, ischemic stroke, or cardiovascular hospitalization support this concern, although residual confounding by disease severity, radioiodine use, surveillance intensity, and baseline risk remains difficult to eliminate (25–28).

### 5.3 Loss of cardiovascular reserve

Metabolically unhealthy survivors may also have reduced myocardial and systemic cardiovascular reserve. Thyroid hormone excess can increase cardiac workload and alter ventricular relaxation, while subclinical thyroid dysfunction may affect cardiac structure and performance even when circulating thyroid hormone levels are not frankly abnormal (38, 39). In DTC-specific mechanistic studies, long-term levothyroxine suppression has been associated with impaired arterial elasticity and increased left ventricular mass, and thyroid hormone perturbation has been shown to affect myocardial strain (23, 24). These findings support the possibility that chronic suppression may influence subclinical cardiovascular remodeling before overt events occur.

The clinical expression of this remodeling is likely modified by age and comorbidity. A young survivor without hypertension, diabetes, obesity, renal disease, or coronary disease may compensate for modest increases in heart rate and myocardial workload. In contrast, an older survivor with central obesity, hypertension, diabetes, dyslipidemia, chronic kidney disease, or subclinical coronary disease may have less physiological reserve. In this setting, the same TSH target may produce a larger clinical burden, especially after years of cumulative exposure to suppressive levothyroxine therapy (27, 28).

**Figure 1:**
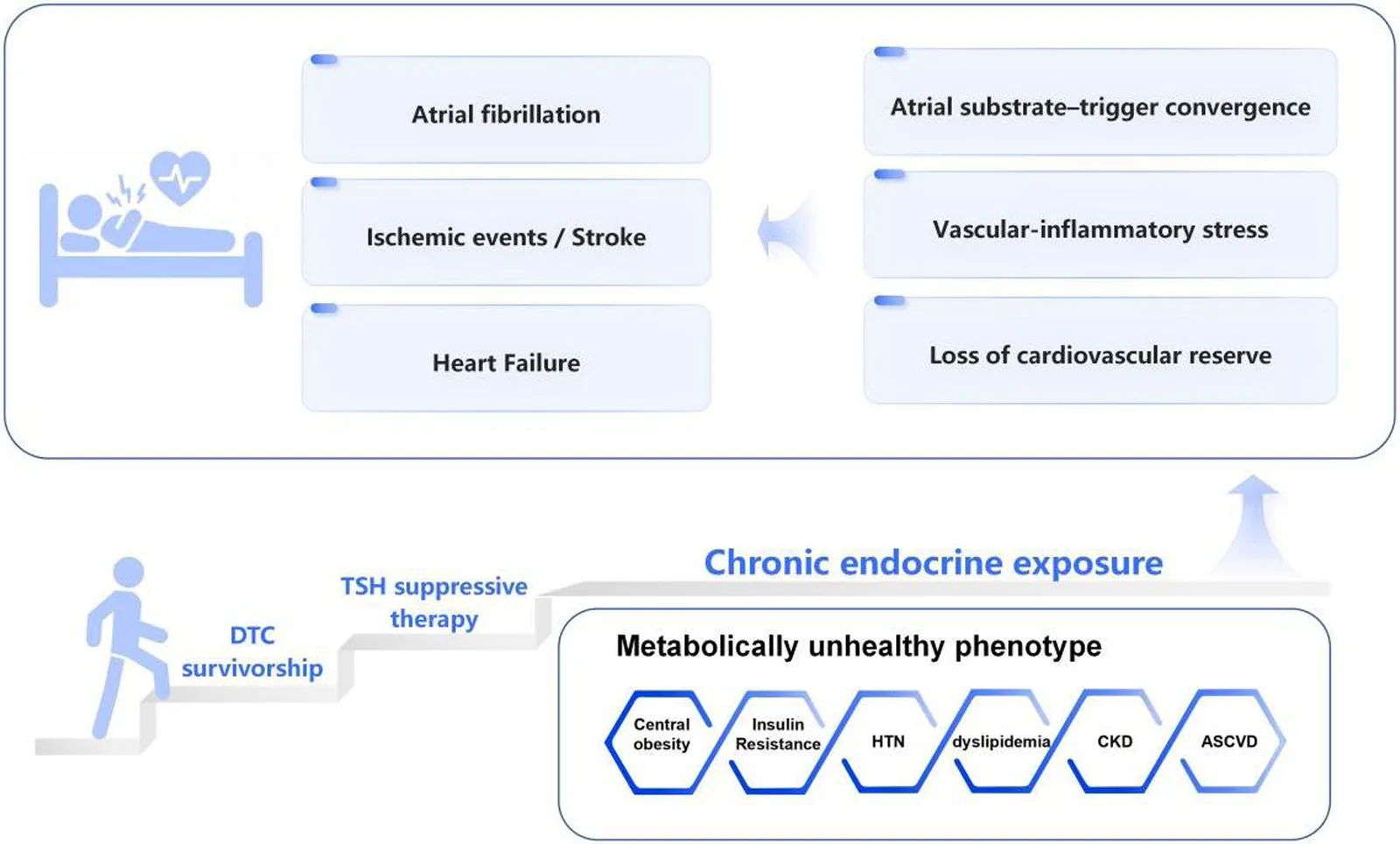
Proposed pathophysiological framework linking long-term TSH suppression, cardiometabolic dysfunction, and cardiovascular vulnerability in differentiated thyroid cancer survivorship. Long-term thyroid-stimulating hormone (TSH)-suppressive therapy in differentiated thyroid cancer (DTC) survivors produces a chronic endocrine exposure characterized by low or suppressed TSH and, in many patients, an exogenous subclinical hyperthyroidism-like state. The treatment sequence from DTC survivorship to suppressive levothyroxine therapy, together with the recognized effects of thyroid hormone excess on adrenergic tone, heart rate, myocardial workload, and hemodynamic stress, reflects established clinical practice and thyroid–cardiovascular physiology. Central obesity, insulin resistance, hypertension, dyslipidemia, chronic kidney disease, and established atherosclerotic cardiovascular disease represent established markers of baseline cardiometabolic vulnerability. Associations of DTC treatment or TSH suppression with atrial fibrillation and other cardiovascular outcomes are supported mainly by observational studies and do not establish causality. By contrast, the interaction between chronic endocrine exposure and cardiometabolic vulnerability, including atrial substrate–trigger convergence, vascular-inflammatory stress, and loss of cardiovascular reserve, represents a proposed and indirectly supported mechanistic framework. Whether these interactions causally increase susceptibility to atrial fibrillation, ischemic events or stroke, heart failure, and cardiovascular mortality in DTC survivors with cardiometabolic dysfunction has not been directly demonstrated. The figure should therefore be interpreted as a hypothesis-generating model rather than a validated causal pathway.

These observations provide biological plausibility for studying a metabolism-informed approach to TSH suppression. Dynamic risk stratification can identify survivors whose recurrence risk has declined after an excellent response, while longitudinal cardiometabolic assessment can identify those whose cardiovascular vulnerability has increased (12–14, 37). In survivors with low recurrence risk or durable excellent response, relaxation of strong suppression may be considered when permitted by current oncologic guidance. However, metabolic phenotype itself has not been prospectively validated as an indication for changing TSH targets. The convergence described here should therefore be interpreted as a proposed mechanistic model rather than a demonstrated clinical interaction (4, 17). These established physiological effects, observational associations, and proposed mechanistic interactions are summarized in Figure 1.

### 5.4 Peripheral T4-to-T3 conversion and tissue thyroid hormone signaling

Triiodothyronine (T3) is the principal biologically active thyroid hormone in the cardiovascular system, whereas a substantial proportion of circulating and tissue T3 is generated through peripheral deiodination of T4 (38, 39). Chronic inflammation and oxidative stress may alter this conversion. In an LPS-exposed HepG2 model, inflammatory stimulation reduced hepatic DIO1 expression, while a nutraceutical combination restored DIO1 mRNA and protein abundance (45). This experiment provides evidence that inflammatory signaling can influence deiodinase-related pathways, but it did not evaluate DTC, suppressive levothyroxine therapy, direct T4-to-T3 conversion rates, or cardiovascular outcomes. Whether chronic metabolic inflammation alters peripheral thyroid hormone activation in DTC survivors therefore remains a mechanistic hypothesis.

Circulating thyroid hormone concentrations may also incompletely represent tissue-specific thyroid hormone exposure. A liquid chromatography-tandem mass spectrometry method has demonstrated that T3 and T4 can be quantified directly in human and animal myocardial tissue and that plasma-tissue relationships may vary under some experimental conditions (46). However, myocardial T3 has not been systematically measured in DTC survivors receiving suppressive levothyroxine. It is therefore unknown whether patients with similar serum TSH and FT4 values have clinically meaningful differences in myocardial T3 availability or signaling.

## 6 Treatment-related modifiers that complicate cardiovascular risk assessment

### 6.1 Radioiodine exposure and thyroid hormone perturbation

Radioiodine therapy should be interpreted as a treatment-related cardiovascular modifier rather than as the central driver of cardiovascular risk in most DTC survivors. In contemporary DTC care, RAI use is increasingly individualized according to recurrence risk, postoperative disease burden, thyroglobulin status, imaging findings, and expected treatment benefit (1, 2). This is important because patients selected for RAI may differ systematically from those not receiving RAI, including differences in disease stage, cumulative treatment burden, TSH targets, surveillance intensity, and duration of levothyroxine exposure.

Available nationwide cohort evidence does not consistently support an independent excess cardiovascular risk from RAI itself. In a Korean nationwide cohort study, cardiovascular outcomes including ischemic stroke, ischemic heart disease, hemorrhagic stroke, cerebrovascular disease, and heart failure were not significantly increased among thyroid cancer patients who received RAI compared with those who did not (47). Similarly, a Taiwanese nationwide cohort study found that RAI treatment was not associated with increased long-term cardiovascular morbidity or mortality, although the authors still emphasized the need for cardiovascular risk assessment during follow-up, especially in patients receiving higher cumulative activity (48).

These findings suggest that RAI should not be simplistically framed as a direct cardiovascular toxin in DTC survivorship. However, RAI remains relevant to cardiovascular risk assessment because it often coexists with other risk-modifying factors, including more advanced initial disease, stronger or longer TSH suppression, thyroid hormone withdrawal or recombinant TSH preparation, repeated treatment courses, and closer medical surveillance. For metabolically unhealthy survivors, the key issue may therefore be less about RAI as an isolated exposure and more about the cumulative endocrine and treatment context in which RAI is delivered (47, 48).

### 6.2 Short-term hypothyroidism and metabolic instability

Thyroid hormone withdrawal before RAI therapy can produce an acute severe hypothyroid state, which may transiently disturb cardiometabolic homeostasis. In patients with DTC undergoing RAI therapy, thyroid hormone withdrawal significantly aggravated most lipid parameters, particularly in those with a greater number of metabolic syndrome components, while also producing changes in insulin resistance markers, inflammatory or vascular markers, homocysteine, and renal-related parameters that were largely reversed after levothyroxine reinstitution (49). These data support the concept that thyroid hormone perturbation can unmask or amplify metabolic vulnerability even over a short period.

This short-term hypothyroid phase has particular relevance for metabolically unhealthy survivors. Patients with central obesity, diabetes, dyslipidemia, hypertension, chronic kidney disease, or metabolic syndrome may have less metabolic flexibility and may experience greater lipid, vascular, or renal fluctuation during thyroid hormone withdrawal. Although the available evidence does not prove that transient withdrawal-induced metabolic changes translate into long-term cardiovascular events, it highlights that DTC treatment can involve alternating periods of suppression, withdrawal, and replacement rather than a stable endocrine state (49).

Recombinant human TSH offers an alternative method of TSH stimulation that can avoid the symptomatic hypothyroid state caused by thyroid hormone withdrawal. A multicenter study comparing recombinant human thyrotropin with thyroid hormone withdrawal for detection of thyroid remnant or cancer showed that recombinant human TSH could stimulate radioiodine uptake and thyroglobulin levels without requiring hypothyroid withdrawal (50). For metabolically unhealthy survivors, the choice between withdrawal and recombinant TSH preparation should therefore be considered not only through oncologic adequacy and cost, but also through short-term cardiometabolic tolerance.

### 6.3 Advanced DTC therapy and layered cardiovascular toxicity

Although most DTC survivors do not require systemic therapy, patients with progressive radioiodine-refractory DTC may receive multikinase inhibitors, creating an additional cardiovascular risk layer. In the phase 3 SELECT trial, lenvatinib significantly improved progression-free survival compared with placebo in radioiodine-refractory thyroid cancer, but treatment-related adverse effects were common, and hypertension was among the most frequent toxicities (51). Therefore, in advanced DTC, cardiovascular risk is shaped not only by TSH suppression and metabolic phenotype, but also by antiangiogenic therapy.

Hypertension is the most clinically important cardiovascular toxicity of lenvatinib in this setting. An exploratory SELECT analysis specifically evaluated treatment-emergent hypertension and its relationship with efficacy and safety, reinforcing hypertension as a common and management-relevant event during lenvatinib therapy (52). This is mechanistically plausible because VEGF-pathway inhibition can reduce nitric oxide bioavailability, increase vascular resistance, and promote endothelial dysfunction, effects that may be more problematic in patients with pre-existing hypertension, diabetes, obesity, renal disease, or atherosclerotic disease.

For patients with radioiodine-refractory DTC and high baseline cardiovascular risk, expert reviews emphasize prevention, early detection, and active management of hypertension, renal toxicity, proteinuria, and other vascular complications during multikinase inhibitor therapy (53). More recent thyroid cancer data also indicate that higher baseline blood pressure is a significant risk factor for lenvatinib-induced hypertension, with blood pressure elevation appearing early after treatment initiation (54). These findings strengthen the broader argument of this review: cardiovascular risk in DTC survivorship is cumulative and treatment-layered. TSH suppression should therefore be evaluated alongside metabolic phenotype, prior RAI-related thyroid hormone perturbation, systemic therapy exposure, and baseline cardiovascular reserve.

## 7 A proposed metabolism-informed framework for TSH suppression and cardiovascular survivorship care

Current ATA guidance bases long-term TSH management principally on disease status, recurrence risk, response to therapy, and the balance between expected benefit and treatment-related harm (1, 2). Current cardiovascular evidence consists predominantly of observational DTC studies, meta-analyses of heterogeneous cohorts, and extrapolation from subclinical hyperthyroidism; it does not establish that metabolic phenotype modifies suppression-related cardiovascular risk (4, 6–8). The framework presented below represents the authors’ hypothesis for integrating cardiometabolic vulnerability into survivorship assessment. It is not currently endorsed as a guideline-based method for selecting TSH targets.

### 7.1 Integrating recurrence risk, treatment response, metabolic phenotype, and cardiovascular vulnerability

We propose that the long-term benefit-harm assessment of TSH suppression should consider four interacting domains: initial recurrence risk, dynamic response to therapy, cardiometabolic phenotype, and cardiovascular vulnerability. Initial recurrence risk and response to therapy are established components of guideline-based oncologic decision-making. Cardiometabolic phenotype and cardiovascular vulnerability, by contrast, are proposed markers of treatment tolerance rather than validated determinants of TSH targets. Direct evidence that these factors modify the cardiovascular effects of suppression is currently lacking (1, 2, 12–14).

From a practical perspective, assessment may proceed in three sequential steps. First, the current oncologic need for suppression should be established from structural disease status, biochemical response, thyroglobulin trajectory, imaging findings, and dynamic response to therapy. Second, established cardiovascular disease and cardiometabolic vulnerability should be assessed. Third, these domains should be considered together: when the oncologic indication remains strong, cardiovascular risk should primarily prompt intensified prevention and surveillance rather than automatic relaxation of suppression; when oncologic benefit has become limited after a durable excellent response, high cardiovascular vulnerability may support consideration of a less suppressive target if oncologically permissible. This sequence represents a proposed clinical interpretation rather than a validated decision algorithm. The need for cardiovascular integration is also consistent with modern cardio-oncology principles. The 2022 ESC cardio-oncology guideline emphasizes baseline cardiovascular risk assessment, monitoring during and after cancer therapy, and prevention or treatment of cardiovascular complications in cancer survivors (55). Although DTC is not usually grouped with highly cardiotoxic malignancies, long-term TSH suppression, thyroid hormone perturbation, radioiodine-related treatment context, and tyrosine kinase inhibitor exposure create a cardio-endocrine survivorship scenario in which oncology, endocrinology, and cardiovascular medicine overlap.

A practical framework can therefore divide DTC survivors into clinically distinct groups. Low recurrence risk, excellent response, and metabolically healthy status generally support avoidance of unnecessary strong suppression. Low recurrence risk, excellent response, and metabolic unhealthiness should prompt particular caution, because the expected oncologic benefit of strong suppression may be small while cardiovascular vulnerability is high. Intermediate-risk or indeterminate-response patients with metabolic unhealthiness require individualized TSH targets and repeated reassessment. High-risk or structural incomplete-response patients may still require stronger suppression, but cardiovascular protection should be integrated from the beginning rather than added only after complications occur (4, 17–22).

### 7.2 Cardiovascular surveillance as part of DTC follow-up

Cardiovascular surveillance in DTC should begin with conventional risk assessment rather than disease-specific testing alone. The 2021 ESC cardiovascular prevention guideline emphasizes risk stratification, blood pressure control, lipid management, diabetes prevention or treatment, smoking cessation, lifestyle intervention, and individualized prevention intensity according to total cardiovascular risk (56). These principles are directly applicable to metabolically unhealthy DTC survivors, in whom TSH suppression may interact with hypertension, dyslipidemia, diabetes, obesity, and established atherosclerotic disease. Region-appropriate cardiovascular risk tools, such as SCORE2 or SCORE2-OP in European practice and the pooled cohort equations in relevant North American populations, may be used to contextualize baseline atherosclerotic risk. However, no SCORE2, pooled cohort equation, or other ASCVD risk threshold has been validated as an indication for relaxing TSH suppression in DTC. These scores should therefore support, rather than determine, endocrine decision-making.

At a minimum, baseline and longitudinal assessment should include blood pressure, BMI or waist circumference, fasting glucose or HbA1c, lipid profile, smoking status, renal function, history of coronary artery disease or stroke, heart failure symptoms, prior atrial fibrillation, and family history of premature cardiovascular disease. The 2019 ACC/AHA primary prevention guideline similarly emphasizes comprehensive risk-factor assessment, lifestyle intervention, team-based care, and individualized pharmacological prevention for atherosclerotic cardiovascular disease (57). For DTC survivors, these assessments should be interpreted together with TSH trajectory, levothyroxine dose, suppression duration, treatment response, and recurrence risk.

Rhythm surveillance deserves particular attention because atrial fibrillation is the most consistent cardiovascular signal associated with thyroid hormone excess and DTC survivorship. The 2023 ACC/AHA/ACCP/HRS atrial fibrillation guideline emphasizes AF risk-factor modification and management of comorbidities, including obesity, hypertension, diabetes, sleep-disordered breathing, and other cardiovascular conditions (58). In metabolically unhealthy DTC survivors, ECG assessment should be considered in older patients, symptomatic patients, those with prolonged strong TSH suppression, and those with hypertension, diabetes, obesity, sleep apnea, structural heart disease, or prior arrhythmia. This recommendation is supported by DTC-specific meta-analyses and cohort studies linking DTC treatment or TSH suppression with increased AF risk (18–22). In this proposed framework, established atrial fibrillation, heart failure, atherosclerotic cardiovascular disease, clinically important arrhythmia, or structural heart disease should carry greater clinical weight than metabolic syndrome alone, because they represent existing cardiovascular disease rather than future risk markers.

### 7.3 Therapeutic implications and risk-factor intervention

A metabolism-informed assessment should not independently determine TSH targets, but it may prompt closer reassessment of whether the intensity and duration of suppression remain justified by the patient's current oncologic status. In low-risk or durable excellent-response survivors with cardiometabolic dysfunction, clinicians may reconsider continued strong suppression when current recurrence risk and response to therapy permit a less suppressive target (1, 2, 4, 17).

Cardiovascular prevention should proceed in parallel with TSH target reassessment. Lifestyle intervention, weight management, blood pressure control, lipid-lowering therapy, diabetes management, smoking cessation, physical activity, and treatment of sleep-disordered breathing are not ancillary issues in this population; they are part of the strategy that determines whether long-term endocrine therapy remains safe. General cardiovascular prevention guidelines provide the framework for these interventions, while cardio-oncology guidance supports coordinated risk assessment and prevention in cancer survivors (55–57).

For patients who still require strong suppression because of high-risk disease or structural incomplete response, cardiovascular risk should primarily prompt active risk mitigation rather than automatic relaxation of suppression. These patients may require more frequent blood pressure monitoring, periodic ECG assessment, aggressive management of dyslipidemia and diabetes, evaluation for ischemic or heart failure symptoms, and a lower threshold for cardiology referral. In survivors with cardiometabolic dysfunction and a durable excellent response, a less suppressive target may be considered when permitted by current oncologic status, together with intensified cardiovascular prevention. The aim is to avoid suppression beyond the level required for oncologic safety while actively managing modifiable cardiovascular risk. A single treatment protocol is unlikely to be appropriate for all DTC survivors because the oncologic consequences of relaxing suppression differ substantially between durable excellent response and persistent structural disease. The proposed framework therefore prioritizes current oncologic status and does not define a universal TSH, FT3/FT4, BNP, or ASCVD-risk threshold. Table 2 summarizes this proposed approach and should not be interpreted as a validated treatment algorithm.

**Table 2:** Proposed metabolism-informed framework for TSH strategy and cardiovascular surveillance in DTC survivors.

Survivor phenotype	TSH strategy	Cardiovascular assessment	Management focus	References
Low-risk + excellent response + metabolically healthy	Avoid unnecessary strong suppression	Routine BP, lipids, glucose/HbA1c, BMI/waist	Standard follow-up; lifestyle prevention	(1, 2, 12–14)
Low-risk + excellent response + metabolically unhealthy	Reassess the ongoing need for strong suppression; consider a less suppressive target only when permitted by current oncologic status	BP, lipids, HbA1c, renal function, ASCVD/AF history	Intensify cardiovascular prevention; avoid suppression beyond the level required by current oncologic status	(4, 17, 29, 30, 55–58)
Intermediate-risk or indeterminate response + metabolically healthy	Individualized moderate suppression	Routine cardiovascular risk assessment	Reassess according to Tg trend and imaging	(1, 2, 12–14)
Intermediate-risk or indeterminate response + metabolically unhealthy	Individualize according to dynamic response; cardiometabolic risk may inform surveillance but does not independently determine the TSH target	Add ECG if older, symptomatic, obese, diabetic, hypertensive, or prolonged suppression	Shared endocrine-cardiology decision-making	(6–8, 18–22, 55–58)
Biochemical incomplete response	Suppression may be justified; adjust by Tg trajectory and risk profile	ASCVD risk, AF risk, BP, lipids, HbA1c	Maintain surveillance; reduce modifiable CV risk	(1, 2, 12, 55–58)
Structural incomplete response or high-risk persistent disease	Stronger suppression may remain necessary	ECG, BP, lipids, HbA1c, renal function, ischemic/HF symptoms	Continue oncologic suppression with active CV protection	(1, 2, 6–8, 18–22)
Established ASCVD, AF, HF, CKD, or older age	Avoid suppression beyond the level required by current oncologic status	ECG/rhythm assessment, BP, renal function, polypharmacy	Individualized TSH target; low threshold for cardiology referral	(21, 22, 25–30, 55–58)
RAI preparation with thyroid hormone withdrawal	Consider short-term metabolic tolerance	Lipids, glucose, BP, renal function	Consider rhTSH when appropriate; monitor metabolic fluctuation	(47–50)
RAIR-DTC receiving VEGFR-TKI	Evaluate TSH strategy with TKI vascular toxicity	Frequent BP, renal function, proteinuria, thrombotic/ischemic symptoms	Early BP control; cardio-oncology input if high risk	(51–55)

These considerations integrate guideline-based oncologic principles, general cardiovascular prevention recommendations, and indirect evidence from DTC and subclinical hyperthyroidism studies; they have not been validated as a DTC-specific decision algorithm. TSH targets should remain anchored to current disease status and response to therapy, while cardiometabolic factors may inform surveillance, cardiovascular prevention, and risk mitigation rather than independently determine treatment intensity. Suggested surveillance may include blood pressure assessment at each routine clinical visit; fasting glucose or HbA1c, lipid profile, and renal function at baseline and at least annually; and baseline plus periodic ECG assessment, approximately annually in older or high-risk patients and in those receiving prolonged strong suppression. Earlier ECG or ambulatory rhythm monitoring should be considered in patients with palpitations, unexplained dyspnea, persistent tachycardia, or an irregular pulse. Cardiology referral should be considered for established or newly detected atrial fibrillation, heart failure, atherosclerotic cardiovascular disease, clinically significant ECG abnormalities, persistent tachycardia, or uncontrolled hypertension. No single metabolic abnormality, cardiovascular risk score, or monitoring interval independently mandates modification of oncologically indicated TSH suppression. AF, atrial fibrillation; ASCVD, atherosclerotic cardiovascular disease; BMI, body mass index; BP, blood pressure; CKD, chronic kidney disease; CV, cardiovascular; DTC, differentiated thyroid cancer; ECG, electrocardiography; HF, heart failure; RAI, radioactive iodine; RAIR-DTC, radioiodine-refractory differentiated thyroid cancer; rhTSH, recombinant human TSH; Tg, thyroglobulin; TKI, tyrosine kinase inhibitor; TSH, thyroid-stimulating hormone; VEGFR, vascular endothelial growth factor receptor.

## 8 Unresolved questions and research agenda

### 8.1 Evidence gaps in current survivorship research

Despite increasing interest in cardiovascular outcomes among DTC survivors, the current evidence base remains limited by observational design, heterogeneous patient selection, incomplete exposure measurement, and variable adjustment for confounders. Many studies do not contain longitudinal TSH trajectories, free thyroxine levels, levothyroxine dose intensity, suppression duration, treatment response, recurrence status, or detailed cardiometabolic phenotyping. As a result, it remains difficult to separate the effect of TSH suppression itself from the effects of age, disease severity, radioiodine exposure, systemic therapy, surveillance intensity, and baseline cardiovascular risk (4, 6–8). Residual confounding may therefore reflect characteristics of patients requiring prolonged suppression rather than the biological effect of suppression alone.

A second limitation is that many available studies treat DTC survivors as a relatively homogeneous group, although their oncologic and cardiovascular risk profiles differ substantially. A low-risk patient with excellent response, mild TSH suppression, and no metabolic disease is biologically and clinically distinct from an older patient with structural incomplete response, strong long-term suppression, diabetes, hypertension, dyslipidemia, and prior coronary disease. Future studies should therefore avoid collapsing these phenotypes into a single exposure category, because doing so may dilute both oncologic benefit and cardiovascular harm signals (12–14, 18–22).

Current cardio-oncology risk tools also do not fully address the specific cardio-endocrine context of DTC. The HFA-ICOS position statement provides practical baseline cardiovascular risk assessment tools for patients scheduled to receive cardiotoxic cancer therapies, but these tools were primarily developed around therapies such as anthracyclines, HER2-targeted therapy, VEGF inhibitors, and other systemic treatments rather than long-term suppressive levothyroxine exposure (59). DTC survivorship therefore requires a more tailored research agenda that incorporates both cancer-treatment exposures and thyroid hormone biology.

### 8.2 Need for phenotype-stratified and longitudinal studies

Future studies should test whether metabolic phenotype modifies the cardiovascular harm or oncologic benefit of TSH suppression. Key variables should include age, sex, menopausal status, obesity phenotype, waist circumference, diabetes status, insulin resistance, blood pressure, lipid profile, chronic kidney disease, atrial fibrillation history, baseline ASCVD, sleep-disordered breathing, TSH trajectory, free thyroxine level, levothyroxine dose, and duration of suppression. This design would allow investigators to determine whether the same TSH target has different clinical implications in metabolically healthy and metabolically unhealthy survivors (29, 30, 37).

The importance of survivor-specific cardiovascular research is supported by broader oncology evidence. A UK Biobank cohort study and meta-analysis reported increased cardiovascular disease risk among cancer survivors, reinforcing the need for routine cardiovascular risk-factor assessment in survivorship care (60). Although thyroid cancer differs from malignancies treated with highly cardiotoxic chemotherapy or radiotherapy, the same survivorship principle applies: as cancer-specific mortality decreases, competing cardiovascular morbidity and mortality become increasingly relevant to long-term outcomes.

Longitudinal designs are particularly important because both DTC recurrence risk and metabolic health change over time. A patient may move from intermediate recurrence risk to excellent response after several years, while simultaneously moving from metabolic health to diabetes, hypertension, dyslipidemia, or subclinical cardiovascular disease. Therefore, future DTC cohorts should measure not only baseline exposures but also time-updated TSH, metabolic status, treatment response, and cardiovascular outcomes. Without this approach, studies may misclassify both the benefit and harm of long-term suppression.

### 8.3 Toward trial-ready clinical questions

The most clinically relevant question is whether relaxing TSH targets in metabolically unhealthy excellent-response survivors can reduce atrial fibrillation, ischemic events, heart failure, or cardiovascular mortality without increasing recurrence. Such a trial would require careful selection of patients with low recurrence risk or durable excellent response, standardized TSH target ranges, serial thyroglobulin and imaging surveillance, adjudicated cardiovascular endpoints, and predefined safety stopping rules for biochemical or structural recurrence. Existing evidence supports equipoise because the oncologic benefit of strong suppression is uncertain in selected survivors, while cardiovascular harm remains plausible (4, 16, 17).

Risk prediction is another trial-ready research area. In the ARIC study, the pooled cohort equations overestimated ASCVD risk in both cancer survivors and cancer-free participants and showed poor discrimination, although performance was broadly similar between groups (61). This finding suggests that conventional cardiovascular risk tools may still be useful starting points, but they are unlikely to capture DTC-specific variables such as TSH depth, levothyroxine exposure, treatment response, thyroid hormone withdrawal, or radioiodine-refractory therapy. Future models should therefore test whether adding DTC-specific endocrine and oncologic variables improves prediction beyond standard ASCVD risk factors.

Finally, prediction-model development should proceed cautiously. A recent systematic review and meta-analysis of cancer therapy-related cardiac dysfunction prediction models found that many models have been developed, but limitations in validation, bias, and generalizability remain important barriers to clinical adoption (62). DTC-specific cardio-endocrine models should therefore be externally validated, interpretable, and clinically actionable, rather than simply statistically complex. The long-term goal should be a practical decision tool that identifies which survivors can safely relax suppression, which require continued suppression with intensified cardiovascular prevention, and which need cardio-endocrine co-management from the beginning.

Future studies should determine whether circulating indices of peripheral thyroid hormone activation, including FT3, FT4, and the FT3/FT4 ratio, provide information beyond TSH for cardiovascular phenotyping in DTC survivors. Low T3 and reduced FT3/FT4 ratios have been associated with adverse outcomes, nutritional vulnerability, or frailty in non-DTC populations (63, 64). These findings support biological interest but do not establish that FT3/FT4 should guide suppressive therapy. Prospective DTC studies should therefore evaluate whether these measures are reproducible, independent of age, nutrition, inflammation, renal function, and illness severity, and incrementally predictive of arrhythmia, heart failure, or treatment intolerance.

Natriuretic peptides may also warrant investigation in this context. Experimental data suggest a relationship between myocardial thyroid hormone signaling and BNP expression, while clinical studies in heart failure populations have reported inverse associations between circulating FT3 and BNP (65, 66). However, BNP and NT-proBNP are strongly influenced by age, renal function, atrial fibrillation, ventricular loading conditions, and established heart failure. They cannot currently be regarded as surrogate measures of myocardial T3 content or as validated biomarkers for titrating levothyroxine in DTC. Future studies should test whether FT3/FT4 and natriuretic peptides provide incremental information beyond conventional thyroid tests and established cardiovascular assessment.

## 9 Conclusion

Long-term TSH suppression in differentiated thyroid cancer should be understood as a dynamic benefit-harm intervention rather than a uniform survivorship routine. Its oncologic value remains most defensible in persistent high-risk disease, biochemical incomplete response, or structural incomplete response, whereas the incremental benefit of prolonged strong suppression becomes less certain in low-risk or durable excellent-response survivors. Obesity, insulin resistance, diabetes, hypertension, dyslipidemia, chronic kidney disease, atrial fibrillation, heart failure, and established atherosclerotic cardiovascular disease identify patients with reduced cardiovascular reserve, but direct evidence that these phenotypes modify the cardiovascular effects of TSH suppression is lacking. Current observational evidence supports careful reassessment and cardiovascular prevention, not causal attribution or a universal mandate to relax suppression. The metabolism-informed framework proposed in this review therefore integrates oncologic status, dynamic response, cardiometabolic vulnerability, cumulative exposure, and cardiovascular reserve as a hypothesis-generating model. Prospective phenotype-stratified cohorts and intervention studies are required before this approach can be translated into validated TSH targets or clinical decision rules.
